# Development of diagnostic algorithm for Cushing’s syndrome: a tertiary centre experience

**DOI:** 10.1007/s40618-024-02354-x

**Published:** 2024-03-27

**Authors:** A. Efthymiadis, H. Loo, B. Shine, T. James, B. Keevil, J. W. Tomlinson, A. Pal, R. Pofi

**Affiliations:** 1 Oxford Centre for Diabetes, Endocrinology and Metabolism, NIHR Oxford Biomedical Research Centre, University of Oxford, Churchill Hospital, Oxford, UK; 2 Department of Clinical Biochemistry, Oxford University Hospitals NHS Foundation Trust, Oxford, UK; 3 Department of Clinical Biochemistry, Manchester University Foundation Trust, Manchester Academic Health Sciences Centre, Manchester, UK

**Keywords:** Late night salivary cortisol, Late night salivary cortisone, Overnight dexamethasone test, 24 h urinary free cortisol, Cushing’s Syndrome

## Abstract

**Purpose:**

No consensus exists as the gold standard for Cushing’s Syndrome (CS) screening. This study aimed to evaluate the diagnostic accuracy and utility of late-night salivary cortisol (LNSC) and cortisone (LNSE), overnight dexamethasone suppression test (ODST), and urinary free cortisol (UFC) in developing a screening algorithm for CS.

**Methods:**

A retrospective, single-centre analysis on 93 adult patients referred to the Oxford Centre for Diabetes, Endocrinology, and Metabolism for CS evaluation (2017–2022). Data were analysed using binomial logistic regression and area under the receiver-operating curve (AUROC).

**Results:**

Fifty-three patients were diagnosed with CS. LNSC (sensitivity 87.5%, specificity 64.9%, AUC 0.76), LNSE (sensitivity 72.4%, specificity 85.7%, AUC 0.79), and ODST (sensitivity 94.7%, specificity 52.1%; AUC 0.74) demonstrated comparable effectiveness for CS diagnosis. Their combined application increased diagnostic accuracy (AUC 0.91). UFC was not statistically significant. Pre-test clinical symptom inclusion improved screening test performance (AUC LNSC: 0.83; LNSE: 0.84; ODST: 0.82). For CD diagnosis, LNSE + LNSC (AUC 0.95) outperformed ODST. Combining these with ACTH levels < 12.6 pmol/L perfectly distinguished MACS (AUC 1.00). ODST (AUC 0.76) exhibited superior performance (sensitivity 100.0%, specificity 52.2%) in MACS detection.

**Conclusions:**

LNSC, LNSE, and ODST are robust tools for CS screening, with their combined use offering the highest diagnostic precision. LNSE, especially when used with LNSC, is highly effective for CD diagnosis, exceeding ODST accuracy. ODST is preferable for MACS identification. Integrating ACTH levels markedly improves differentiation between CD and MACS. Conversely, UFC shows limited diagnostic utility.

**Supplementary Information:**

The online version contains supplementary material available at 10.1007/s40618-024-02354-x.

## Introduction

Endogenous Cushing Syndrome (CS) refers to pathologic hypercortisolism and is associated with significant morbidity, mortality, and reduction in quality of life [1–4]. The diagnosis of CS can often be challenging [5] as it is typically characterized by the presence of multiple symptoms (such as hypertension, diabetes, weight gain, or osteoporosis) which are very common in the general population. Clinical pre-test likelihood of CS should be evaluated [6] and screening conducted in those with either signs or symptoms of low discriminatory value (e.g., hypertension and osteoporosis) occurring at an unusually early age, or patients with clinical features of higher specificity for CS (e.g., easy bruising, facial plethora, proximal myopathy, and Striae rubrae).

Several diagnostic testing strategies have been proposed, and at least two clinical scores have been recently developed to identify patients deserving screening for CS [7, 8]; however, their value in everyday clinical practice remains uncertain, since validation studies for these scores are missing. To date, there is no consensus as to the gold standard screening test for the diagnosis of CS, and the presence of at least two abnormal tests with high diagnostic accuracy is needed [9, 10].

The decision on which screening test to choose is influenced by a combination of factors, including the index of clinical suspicion for hypercortisolism, the suspected underlying diagnosis, and, importantly, local availability of specific tests. The latest consensus recommended that if CS is suspected any combination of overnight dexamethasone suppression test (ODST), urinary free cortisol (UFC), and late-night salivary cortisol (LNSC) tests can be helpful [9]. For patients with adrenal incidentalomas being evaluated for hypercortisolism, ODST is recommended as a first test, with consideration of additional UFC and/or LNSC measurements [9, 11]. The role of Late-Night Salivary Cortisone (LNSE) as a CS screening is still unclear. Moreover, the cost-effectiveness of various hypercortisolism screening strategies remains a subject of ongoing debate. This is particularly relevant given the increasing frequency of hypercortisolism screenings in low-risk populations (e.g., obese patients) and their use in the diagnostic workup for incidentally discovered adrenal lesions. The challenge lies in identifying a screening strategy that exhibits high diagnostic accuracy and acceptable costs but is simultaneously acceptable from the patient’s perspective.

The aims of this study were: (a) to compare the ability of LNSC, LNSE, ONDT, and UFC as screening test for CS; (b) to evaluate the diagnostic performance of each test in distinguishing Cushing’s disease (CD) or mild autonomous cortisol secretion (MACS) from patients without CS; (c) to suggest a screening algorithm.

## Materials and methods

### Patient selection

We retrospectively reviewed all consecutive adult patients referred at the Oxford Centre for Diabetes, Endocrinology and Metabolism (OCDEM) to be evaluated for hypercortisolism and who had LNSC measured, from January 2017 to November 2022. Reasons to screen for hypercortisolism included an incidental diagnosis of adrenal adenoma, the presence of phenotypic characteristics of CS (i.e., dorsal fat pad, central adiposity, facial plethora, easy bruising, purple striae, hirsutism, and proximal myopathy) as well as combinations of hypertension, insulin-resistant diabetes, oligomenorrhea, osteoporosis, and mood disorders.

### Screening tests

We collected data for the following screening tests: LNSC, LNSE, ODST, UFC, and Low-Dose Dexamethasone Suppression Test (LDDST).

A cut off of 50 nmol/L (1.8 μg/dL) for 9 AM cortisol levels after dexamethasone (DEX) challenge was used to interpret ODST (1 mg of DEX between 23:00–24:00 and subsequent 9am cortisol) and LDDT (0.5 mg of DEX 6-hourly for 48 h with cortisol checked at time 0’ and 48 h). At least two 24-h urine samples were requested to the patients screened through UFC and values less than 135 nmol/L were considered normal. Total urine volume and creatinine clearance were measured to ensure adequate urine collection. Similarly, all the patients screened with LNSC and LNSE had at least two evaluations. Cut-off values of < 1.7 nmol/L for LNSC, < 18.0 nmol/L for LNSE were considered normal based on assay-specific reference ranges [12, 13]. We also collected data on ACTH levels performed as baseline sample for LDDST. In our institution, the latter is used either in lieu of ODST or where results of ODST are equivocal. The diagnosis of CD was confirmed through inferior petrosal sinus sampling and histology. Patients with adrenal lesions and a ODST above the 50 nmol/L cut-off were diagnosed as mild autonomous cortisol secretion (MACS) as per current guidelines [14] by experienced endocrinologists. Subjects in whom CS was ruled out based on the outcomes of the screening tests (ODST, UFC, LNSC, and LNSE) will be referred to as "*controls*" throughout the manuscript. Patients with proven CS will be referred to as "*cases*".

### Data collection

Electronic patient records were reviewed. Patient demographics and anthropometric measurements (i.e., age, sex, weight, and body mass index), relevant comorbidities (i.e., obesity, hypertension, insulin-resistant diabetes, osteoporosis, obstructive sleep apnoea, mood disorders, and hypokalaemia), relevant medications (i.e., steroids, hormonal replacement therapies), clinical signs, and symptoms suggestive of CS (dorsal fat pad, facial plethora, proximal myopathy, purple striae, hyperpigmentation, and fatigue) were collected.

### Laboratory analysis

As described previously, saliva cortisol/cortisone [12, 13] were measured by electrospray positive ion mode liquid chromatography tandem mass spectrometry. The lower limit of quantification was 0.46 nmol/L for salivary cortisol and 0.42 nmol/L for salivary cortisone. Between-batch imprecision for cortisol showed coefficient variations of 13.4% to 2.7% across a range of concentrations from 4.2 to 118 nmol/L. Between-batch imprecision for salivary cortisone showed coefficient variations of 8.6% to 2.3% across a range of concentrations from 5.0 to 130.9 nmol/L. Recovery was 93% and 96% for cortisol and cortisone, respectively. 20 Alpha and 20 beta dihydrocortisone showed baseline separation with cortisone and did not interfere in the assay. Serum cortisol was measured through the Abbott Architect i2000 (Abbott Laboratories, Maidenhead, UK) which had between-batch coefficient of variation of 4.1% at 118 nmol/L, 2.8% at 427 nmol/L and 2.8% at 967 nmol/L. UFC was determined following dichloromethane solvent extraction and liquid chromatography tandem mass spectrometry. Between-batch precision was 3.2% at 144 nmol/L and 2.2% at 692 nmol/L. The lower limit of quantification was 16 nmol/L, and the upper limit of linearity was 1600 nmol/L. ACTH measurements were undertaken using an Immulite 2000 analyser (Siemens Healthineers, Frimley, UK), a solid-phase, two-site sequential chemiluminescent immunometric assay (CLIA). Method imprecision, expressed as CV% was 8.2% at 26.8 pmol/L and 4.9% at 375.7 pmol/L.

### Statistical analysis

Sensitivity, specificity, likelihood ratios, as well as predictive values were computed for each biochemical test (LNSC, LNSE, ODST, UFC, and LDDST). The *χ*^2^ test was used to test statistical significance associations between binomial variables. A *p* < 0.05 was considered indicative of a statistically significant difference. A binomial logistic regression (LR) was performed to ascertain the ability of each test in diagnosing CS, calculate odds ratios (OR) and extract predictive values to compute ROC curve analysis. Comparison between the AUCs of each test was made for patients having both tests (De Long et al.[15]) as well as with independent comparisons. A stepwise backward LR was run to assess the utility of clinical symptoms to predict the diagnosis of CS. Only the symptoms retaining statistically significant association at univariate analysis were included into the model as independent variables (hypertension, dorsal fat pad, facial plethora, striae rubrae, myopathy, and easy bruising). Independent meaningful correlation was defined by a *p* value < 0.05 with the outcome of interest. The extracted predictive value from LR analysis on clinical symptoms was then used to refine the diagnostic accuracy of biochemical tests by combining the relative AUCs. The analysis was then stratified according to disease aetiology (either CD or MACS). ROC curve analysis was use to confirm optimal cut-offs stratified for disease aetiology using the raw data. To evaluate the ability of ACTH levels to predict CD *vs* MACS, the baseline ACTH levels of the LDDST were included into the binomial logistic regression model. Statistical analyses were performed using SPSS (version 29, Chicago, IL, USA), illustrations were made with GraphPad Prism 8.0 software package (GraphPad Software, Inc. La Jolla, CA, USA) and ROC curve comparisons were conducted using MedCalc software Ltd (Ostend, Belgium).

### Ethics

The study was approved by the Trust audit team and conducted and registered as a local audit of practice (reference number 8352).

## Results

A total of 93 subjects were included in the analysis. Fifty-three patients were diagnosed with CS (79% females, mean age 56 ± 16 years, and mean body mass index 31.7 ± 7.2 kg/m^2^), whereas the diagnosis was excluded in 40 subjects (mean age was 51 ± 18 years, 72% females, and mean body mass index 34.8 ± 9.3 kg/m^2^). Among patients with CS, 24 (47%) and 27 (53%) were diagnosed with MACS and CD, respectively. Two patients were diagnosed with ectopic disease and were excluded from the analysis.

### The performance of biochemical screening tests

**Fig. 1:**
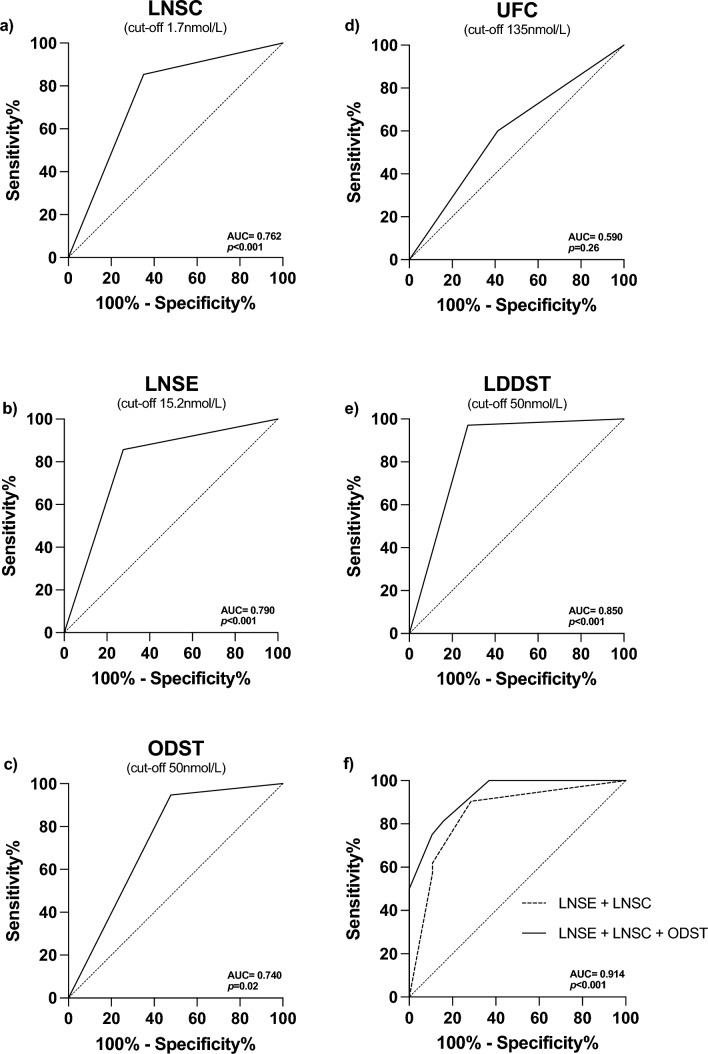
ROC curves of commonly used screening test for the diagnosis of Cushing’s syndrome

Sensitivity and specificity of LNSC in distinguishing *cases* and *controls* were 87.5% (95% CI 73.2–95.8) and 71.1% (95% CI 73.2–95.8), respectively, and the NLR was 0.19, with good diagnostic accuracy (AUC 0.762, 95%CI 0.650–0.873, *p* < 0.001) (Fig. 1a, Table 1). LNSE showed higher specificity (96.1%; 95% CI 80.4–99.9) but lower sensitivity (55.8%; 95% CI 37.9–72.8), a NLR of 0.46, with comparable diagnostic accuracy (AUC 0.791, 95% CI 0.660–0.921, *p* < 0.001) (Fig. 1b, Table 1). The ODST also demonstrated similar performance (AUC 0.740, 95% CI 0.59–0.88; *p* = 0.02), with sensitivity and specificity of 94.7% (95% CI 82.2–99.3) and 52.1% (95% CI 30.6–73.2) respectively, and a NLR of 0.10 (Fig. 1c, Table 1). On the contrary, UFC was not able to reach statistical significance in distinguishing *cases* form *controls* (AUC 0.59, 95% CI 0.43–0.76; *p* = 0.26) (Fig. 1d, Table 1), showing low sensitivity (60.0%, 95% CI 43.3–75.1) and specificity (58%, 95% CI 32.9–81.6).

**Table 1:** The diagnostic accuracy of screening tests stratified for disease aetiology

Test	AUC (95% CI)	Sensitivity (95% CI)	Specificity (95% CI)	Positive likelihood ratio	Negative likelihood ratio	Positive predictive value	Negative predictive value
Cases vs controls							
LNSC	0.76 (0.65–0.87) *p* < 0.001	87.5% (73.2–95.8)	71.1% (73.2–95.8)	2.49	0.19	76.1%	84.3%
LNSE	0.79 (0.66–0.92) *p* < 0.001	55.8% (37.9–72.8)	96.1% (80.4–99.9)	14.53	0.46	95.0%	62.5%
ODST	0.74 (0.59–0.88) *p* = 0.020	94.7% (82.2–99.3)	52.1% (30.6–73.2)	1.98	0.10	76.6%	85.7%
LDDST	0.85 (0.69–1.0) *p* < 0.001	97.4% (86.5–99.9)	72.7% (39.0–94.0)	3.57	0.04	92.7%	88.9%
UFC	0.59 (0.43–0.76) *p* = 0.260	60.0% (43.3–75.1)	58.0% (32.9–81.6)	1.46	0.68	77.4%	38.5%
CD vs controls							
LNSC	0.82 (0.72–0.93) p < 0.001	100% (80.5–100)	64.9% (47.5–79.8)	2.85	0.00	56.7%	100%
LNSE	0.93 (0.83–1.00) *p* < 0.001	86.7% (59.5–98.3)	96.1% (80.4–99.9)	22.53	0.14	92.9%	92.6%
ODST	0.71 (0.55–0.87) p = 0.03	88.9% (65.3–96.6)	52.2% (30.6–73.2)	1.86	0.21	59.3%	85.7%
LDDST	0.86 (0.70–1.0) p = 0.001	100% (84.6–100)	72.7% (39.0–94.0)	3.67	0.00	88.0%	100%
UFC	0.75 (0.58–0.91) p = 0.008	90.9% (70.8–98.9)	58.8% (32.9–81.6)	2.21	0.15	74.1%	83.3%
MACS vs controls							
LNSC	0.71 (0.57–0.85) *p* = 0.007	77.2% (54.6–92.2)	64.8% (47.5–79.8)	2.20	0.3	56.7%	82.8%
LNSE	0.66 (0.47–0.85) *P* = 0.102	27.8% (9.7–53.5)	96.1% (80.4–99.9)	7.22	0.75	83.3%	65.8%
ODST	0.76 (0.66–1.00) p = 0.004	100% (82.4–100.0)	52.2% (30.6–73.2)	2.09	0.00	63.3%	100%
LDDST	0.83 (0.66–1.00) *p* = 0.004	93.8% (69.8–99.8)	72.7% (39.0–94.0)	3.44	0.09	83.3%	88.9%
UFC	0.62 (0.43–0.80) *p* = 0.242	17.7% (3.8–43.4)	58.8% (32.9–81.6)	0.43	1.4	30.0%	41.7%

*LNSC* late-night salivary cortisol, *LNSE* late-night salivary cortisone, *ODST* overnight dexamethasone test, *LDDST* low-dose (2 mg) dexamethasone test, *UFC* 24 h urinary free cortisol. *CD* Cushing’s disease, *MACS* mild autonomous cortisol secretion

Comparison of AUC for LNSC, LNSE, and ODST demonstrated a negligible, non-significant, difference between areas (*p* > 0.05 for all comparisons). However, considering that LNSC and LNSE can be performed at the same time, we combined the two tests’ prediction and found a better performance AUC of 0.85 (95% CI 0.71–0.94, *p* < 0.001) (Fig. 1f) in distinguishing *cases* and *controls* compared to the LNSC alone (delta AUC 0.09, 95% CI 0.01–0.17, *p* = 0.02). Adding ODST prediction to the model further increased diagnostic accuracy (AUC of 0.91, 95% CI 0.77–0.98, *p* < 0.001; delta AUC 0.08, 95% CI 0.01–0.17, *p* = 0.06) with 1.7 nmol/L, 15.2 nmol/L and 50 nmol/L being the best cut-offs for LNSC, LNSE, and ODST, respectively. Interestingly, including the UFC to each test or each combination modelling, results in worsening of the diagnostic performance (data not shown). As expected [16], (being used as second screening test in our centre) LDDST had the highest sensitivity (97.4%, 95% CI 86.5–99.9) and specificity (72.7%, 95% CI 39.0–94.0) and diagnostic accuracy (AUC 0.85, 95% CI 0.69–1.0; *p* < 0.001), with the lowest NLR 0.04 (Table 1, Fig. 1d). However, there were no differences compared to the combined performance of LNSC + LNSE + ODST (*p* = 0.49).

### The importance of pre-test probability

**Fig. 2:**
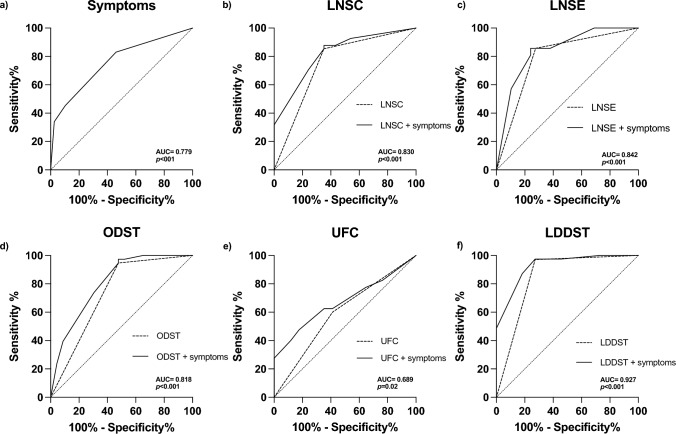
Pre-test probability performance alone and in combination with screening tests for Cushing’s syndrome

Univariate analysis showed hypertension (*χ*^2^ = 10.2, *p* = 0.001), dorsal fat pad (*χ*^2^ = 11.9, *p* < 0.001), facial plethora (*χ*^2^ = 13.0, *p* < 0.001), striae rubrae (*χ*^2^ = 6.2, *p* = 0.013), myopathy (*χ*^2^ = 5.3, *p* = 0.021), and easy bruising (*χ*^2^ = 6.9, *p* = 0.008) as clinical important signs and symptoms associated with the diagnosis of CS. A backward multiple LR analysis was conducted to assess the contribution of each symptom in diagnosing CS (*χ*^2^ = 26.08, *p* < 0.001): hypertension (OR 3.6, 95% CI 1.53–10.21, *p* = 0.005) and facial plethora (OR 6.5, 95% CI 2.10–23.82, *p* = 0.002) where those with strongest prediction for CS (Table 2). ROC curve analysis confirmed good diagnostic performance of pre-test clinical symptoms (AUC 0.779, 95% CI 0.686–0.872, *p* < 0.001) (Fig. 2a). Patients presenting with both hypertension and facial plethora had 19 times higher chance of being diagnosed with CS (OR 19.54, 95% CI 2.47–154.15; *p* < 0.001).

**Table 2:** Backward linear regression modelling of clinical important symptoms in 93 subjects screened for Cushing’s syndrome

	*B*	*p*	Exp (B)	95% confidence interval	
				Lower	Upper
*χ*^2^ = 29.72, *p* < 0.001	Step 1				
Hypertension	1.170	**0.023**	3.223	1.178	8.819
Striae rubrae	0.643	0.519	1.902	0.270	13.404
Facial pletora	1.758	0.069	5.801	0.874	38.483
Dorsal fat pad	0.783	0.354	2.188	0.418	11.449
Easy bruising	0.850	0.236	2.339	0.574	9.540
Myopathy	− 1.038	0.274	0.354	0.055	2.272
Osteopenia	2.078	0.097	7.989	0.685	93.115
*χ*^2^ = 25.81, *p* < 0.001	Step 5				
Hypertension	1.281	**0.005**	3.600	1.372	9.446
Facial pletora	1.875	**0.003**	6.518	1.902	22.332
Osteopenia	1.694	0.091	5.443	0.591	50.139

Significant *p*-values are highlighted in bold

Combining AUC of the pre-test clinical symptoms with those of the screening tests significantly improved diagnostic performance of LNSC (AUC 0.83, 95% CI 0.74–0.92, *p* < 0.001), LNSE (AUC 0.84, 95% CI 0.73–0.95, *p* < 0.001), ODST (0.82, 95% CI 0.71–0.93, *p* < 0.001), and LDDST (AUC 0.93, 95% CI 0.84–1.0, *p* < 0.001). Despite significant improvement in diagnostic performance of UFC with symptoms (AUC 0.689, 95% CI 0.552–0.826, *p* = 0.025), the results still did not reach good diagnostic accuracy (i.e., AUC < 0.7) (Fig. 2b-f).

### Subgroup analysis according to disease aetiology: CD

LNSC had the best sensitivity (100%, 95% CI 80.5–100.0) and NLR (0.00) compared to LNSE (86.7%, 95% CI 59.5–98.3; NLR 0.14) but lower specificity (LNSC 64.9%, 95% CI 47.5–79.8; LNSE 96.1%, 95% CI 80.4–99.9) in distinguishing between patients with CD and those who had hypercortisolism excluded (AUC_LNSC_ 0.82, 95% CI 0.72–0.93, *p* < 0.001; AUC_LNSE_ 0.93, 95% CI 0.83–1.00, *p* < 0.001). However, optimal cut-off derived by ROC analysis revealed lower threshold for LNSE of 15.2 nmol/L as best distinguishing between *CD* and *controls*, with significantly higher sensitivity (100%, 95% CI 78.2–100) and specificity (78.1%, 95% CI 60.0–90.7).

Interestingly, in this context, UFC reached statistical significance (AUC 0.75, 95% CI 0.58–0.91, *p* = 0.008) in distinguishing CD and *controls* with good sensitivity (90.9%, 95% CI 70.8–98.9), low specificity (58.8%, 95% CI 32.9–81.6), and acceptable NLR (0.15). Nevertheless, ODST still retained good sensitivity (88.9%, 95% CI 65.3–96.6) albeit with low specificity (52.2%, 95% CI 30.6–73.2) and NLR (0.21) in diagnosing CD (AUC 0.71, 95% CI 0.55–0.87, *p* = 0.03).

As for the whole cohort, LDDST had high sensitivity (100.0%, 95% CI 84.6–100.0) and specificity (72.7%, 95% CI 39.0–94.0), NLR (0.00) with good diagnostic performance (AUC of 0.86, 95% CI 0.70–1.0, *p* = 0.001). Independent AUCs comparisons showed LNSE to perform better than ODST and UFC in CD. All other comparisons did not show significant differences. As for the whole cohort, combining LNSC and LNSE result in a significant increase in diagnostic accuracy (AUC of 0.95, 95% CI 0.81–1.0, *p* < 0.001), with 1.7 nmol/L and 15.2 nmol/L being the best cut-offs for LNSC and LNSE, respectively.

A summary of ROC analyses for the screening tests in distinguish patients with CD from controls is reported in *supplementary Fig. 1*.

### Subgroup analysis according to disease aetiology: MACS

The ODST was the best test in distinguishing between patients with MACS and those who had hypercortisolism excluded (AUC of 0.76, 95% CI 0.66–1.00, *p* = 0.004) with a sensitivity of 100.0% (95% CI 82.4–100.0), specificity of 52.2% (95% CI 30.6–73.2), and NLR of 0.00. LNSC was not as good as in CD when used to rule out MACS diagnosis: the sensitivity was 77.2% (95% CI 54.6–92.2), the specificity 64.8% (95% CI 47.5–79.8), and NLR 0.3 (AUC 0.711, 95% CI 0.574–0.848, *p* = 0.007). Interestingly, LDDST confirmed high diagnostic performance AUC of 0.83 (95% CI 0.66–1.00, *p* = 0.004) also in this context, albeit with lower sensitivity of 93.8% (95% CI 69.8–99.8), higher specificity of 72.7% (95% CI 39.0–94.0), and NLR of 0.09 when compared to ODST. As per the whole cohort, UFC alone did not distinguish MACS and *controls* (AUC 0.62, 95% CI 0.43–0.80, *p* = 0.242), and the combination with any other test worsens relative diagnostic performance (data not shown). Interestingly, LNSE also did not reach statistical significance (AUC of 0.66, 95% CI 0.47–0.85, *p* = 0.102). There were no differences between the AUCs of the tests at independent comparisons.

A summary of the diagnostic accuracy of the screening tests in distinguish patients with MACS from controls is reported in *supplementary Fig. 2*.

### The significance of ACTH levels in distinguishing CD from MACS

As expected, ACTH levels (median ACTH_MACS_ 8.6 pmol/L, min–max 5.0–25.7 *vs* ACTH_CD_ 57.2 pmol/L, min–max 13.6–273; *p* < 0.001) were lower in patients with MACS as compared with those with CD. Baseline ACTH levels of LDDST were used as dependent variable in a logistic regression model to evaluate its ability in predicting the diagnosis of CD against MACS. The model was significant (*χ*^2^ = 33.78, *p* < 0.001), and ACTH levels were able to localize the disease (*B* = 0.258, *p* = 0.017). An ROC curve analysis (AUC 0.98, 95% CI 0.87–1.00, *p* < 0.001) showed ACTH > 12.6 pmol/L as cut-off distinguishing CD from MACS with 100% sensitivity, 86.7% specificity, and negative likelihood ratio (NLR) of 0.00. Interestingly, combining AUC of the ACTH levels with those of the LNSC + LNSE (as the one with the highest performance in diagnosing CD) improved overall diagnostic performance to an AUC of 1.00 (95% CI 1.00–1.00, *p* < 0.001).

## Discussion

The diagnosis of CS is one of the most challenging in endocrinology. This is the first study comparing the diagnostic performance of five screening test used in the diagnosis of CS with the aim of describing the best combination of tests to be used as a screening strategy and according to disease aetiology. We show that LNSC, LNSE, and ODST individually offer comparable performance in screening for hypercortisolism. However, their combination significantly increases the overall diagnostic performance. In our study, UFC, which is widely used as a first-line screening test in many countries due to its historical application and availability, showed limited diagnostic utility in detecting pathological hypercortisolism when considering all cases collectively. However, the subgroup analysis reveals that whilst UFC demonstrates moderate diagnostic ability in CD, it fails to effectively identify MACS. This outcome aligns with expectations, given that MACS is characterized by subclinical or mild cortisol excess, typically resulting in lower plasma and, consequently, free urinary cortisol levels. This is further reflected by the absence of overt Cushingoid features in MACS patients. Notably, adding the pre-test probability (assessed by clinical symptoms) to biochemical evaluation demonstrated superior diagnostic performance than each separate test alone, and matched the performance of LDDST, a finding not replicated with UFC. Of note, we found that test performance was dependent on aetiology of hypercortisolaemia; in CD, LNSE was most discriminatory whereas for investigation of adrenal incidentaloma and eventual diagnosis of MACS, ODST was the ‘best’ test. When LNSE and ODST are used in combination, the overall diagnostic performance increases significantly. Finally, ACTH levels performed well in confirming CD versus MACS.

Our study emphasises the importance of clinical context when deciding upon if, and how one should screen for Cushing’s syndrome. The existence of clinical features of cortisol excess were clearly associated with diagnosis confirmation (we find hypertension and facial plethora to be independent predictors) and if pre-test probability is moderate to high, the use of LNSC, LNSE, and ODST represents a robust screening approach with 1.7 nmol/L, 15.2 nmol/L, and 50 nmol/L being the best cut-offs for each test, respectively. This challenges the need for the more cumbersome and time-consuming LDDST and UFC. Building upon previous smaller studies that reported increased diagnostic accuracy through the combination of LNSC and LNSE [17], our findings advocate for including ODST to enhance diagnostic precision further. In line with other studies and current guidelines [14, 18], we find that when investigating incidental adrenal lesions, ODST was the superior screening tool. Notably, the specificity of ODST can be further improved by measuring dexamethasone levels. It has been observed that about 6% of patients who do not demonstrate cortisol suppression during ODST actually have suboptimal dexamethasone levels [19]. This underscores the potential benefit of incorporating dexamethasone level measurement in ODST protocols. Regrettably, data on dexamethasone levels were not available in our cohort.

Whilst adrenocorticotropic hormone (ACTH) levels are known to help disease localisation, the optimal ACTH cut-off for distinguishing between adrenal and pituitary CS is still undefined. We found 12.6 pmol/L as the cut-off distinguishing the two aetiologies with high sensitivity and specificity. Previous studies have proposed similar but not identical cut-offs [20, 21], albeit with lower sensitivity and specificity. This variability across studies is likely due to the use of different assays, which hampers the interpretation and comparison of results [22]. Although additional focused research is required to validate our findings, in our cohort, combining ACTH with LNSC and LNSE gave excellent diagnostic accuracy in distinguishing CD from MACS.

Our findings are consistent with the current literature. Updated guidelines recommend the use of at least two screening tests when investigating CS and there is intermediate to high pre-test probability [9], because the diagnostic accuracy increases significantly [23]. A recent systematic review and meta-analysis of 139 studies including 14,140 patients showed that sensitivity and specificity of LNSC, ODST, and UFC for diagnosing CD were close to 90% and, using meta-regression, ODST and UFC were reported as the best and the worst screening tests respectively, albeit the CIs overlapped significantly [24]. We confirmed high sensitivity for all the three tests in diagnosing CD but we found lower specificity, probably due to the lower number of subjects included in the analysis. The fact that our hospital is a tertiary referral centre where subjects have been filtered by primary and secondary care physicians before CS testing could also be contributing to this discrepancy.

Current guidelines emphasise the specificity of LNSC in diagnosing CD. However, as highlighted in previous research [25, 26], LNSC demonstrates limited diagnostic accuracy for adrenal incidentalomas, where the ODST remains the preferred screening tool [14, 27]. Whilst early studies predominantly focused on comparing LNSC with standard screening tests, the diagnostic accuracy of LNSE had been less well described. Recently, other studies reported similar diagnostic accuracy for LNSE in the diagnosis of CD compared with LNSC using LCMS/MS [28, 29] using different cut-offs (13.5–19.9 nmol/L) [30–32]. In our study, using 18 nmol/L as assay-specific cut-off we found sensitivity of 86% and specificity of 96.1% for LNSE. Screening tests should prioritize high sensitivity over specificity [33, 34] and, when using 15.2 nmol/L as optimal cut-off computed by ROC analysis, the sensitivity of LNSE rose to 100%, with minimal effects on specificity.

Our findings indicate that LNSE is the most predictive test for CD, surpassing LNSC in this regard. Supporting our observation, a study focused on CD patients revealed notable fluctuations in LNSC levels over time [31], which might reduce its reliability as a diagnostic tool in certain cases. In contrast, LNSE is derived from the rapid and efficient conversion of free serum cortisol in the salivary glands by the enzyme 11-β-hydroxysteroid dehydrogenase type 2. This process remains effective even when serum cortisol levels are low. Consequently, LNSE generally shows a more consistent correlation with serum cortisol levels compared to LNSC, which can be undetectable at lower concentrations [35, 36]. Yet, from a practical perspective, LNSC and LNSE can be routinely measured simultaneously, and we have shown that measuring both together led to better diagnostic sensitivity than using either test alone in CD.

Nevertheless, like LNSC, LNSE also showed reduced diagnostic accuracy in cases of adrenal hypercortisolism. This discrepancy might stem from the overall lower circulating glucocorticoid levels in MACS compared with full blown CD as well as from their different pattern of fluctuations throughout the day [37], and other researchers showed how peaks in LNSE measurements do not consistently align with elevations detected by other tests [17]. Albeit salivary cortisone is present at a higher concentration than cortisol (cortisone/cortisol ratio 4:1), a retrospective analysis on 173 patients undergoing ODST, LNSC, and LNSE demonstrated that post-ODST cortisone (but not LNSE) was the most significant independent predictor for abnormal ODST, as evidenced in both univariate and multivariate analyses [38]. Whilst our findings align with other studies that confirm LNSC diagnostic accuracy [39–42] and suggest LNSE as a potential tool to enhance the current screening strategy for CS, further research is still needed to clarify the role of LNSE in diagnosing adrenal Cushing’s.

We acknowledge that this study has some limitations and potential biases. First, as a retrospective analysis conducted at a single tertiary referral centre, the findings may not be generalizable to all patient populations. Variations in patient demographics, comorbid conditions, and healthcare settings could influence the performance of the screening tests. Additionally, the retrospective nature of the study introduces the potential for selection bias, as the sample may not represent all patients typically evaluated for CS. Finally, dexamethasone serum levels during ODST were not available in our analysis. Future research should aim to include multicentre, prospective studies to validate these findings across diverse clinical settings and reduce the influence of selection biases.

## Conclusions

**Fig. 3:**
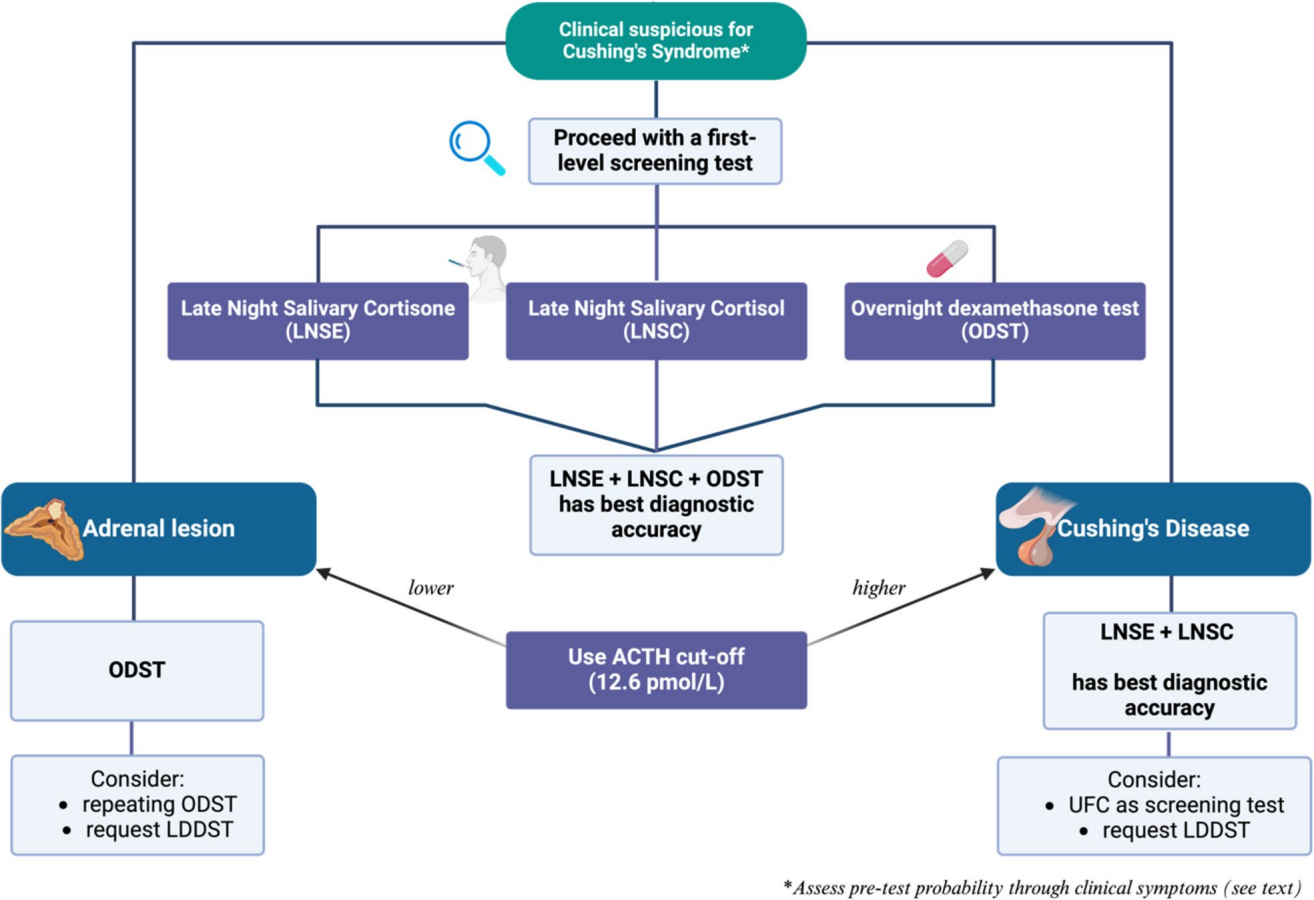
In-house proposed algorithm for Cushing’s syndrome diagnosis

Current guidelines for CS screening remain ambiguous as to the optimal screening approach tailored on patient’s characteristics. Our study is novel in its combination of various screening tests to identify a method with the highest diagnostic precision, rather than assessing each test in isolation. We underscore the superiority of LNSC, LNSE, and ODST over UFC in CS screening and show high diagnostic accuracy comparable to the more time-consuming and labour intensive LDDST. We also stress the value of incorporating clinical probability into the screening, which heighten diagnostic accuracy. We propose a simple screening algorithm based on performance of screening tests in our centre (Fig. 3) which now uses LNSE/C in combination with ODST for screening moderate/high clinical pre-test probability patients, and ODST for adrenal incidentalomas. Our analysis contributes to the current literature examining performance of screening tests in Cushing’s by analysing a sizeable cohort from a single centre, where previously data, particularly on LNSC/E measurement are lacking. This will hopefully inform the development of more precise clinical guidelines in the investigation of CS.

## Supplementary Information

Below is the link to the electronic supplementary material.

Supplementary file1 (DOCX 109 KB)

## Data Availability

The datasets generated during and/or analysed during the current study are available from the corresponding author on reasonable request.
