# Supplementary material for: Development of diagnostic algorithm for Cushing’s syndrome: a tertiary centre experience
Source: J Endocrinol Invest. 2024 Mar 27;47(10):2449–59. doi: 10.1007/s40618-024-02354-x (PMC11393038; doi:10.1007/s40618-024-02354-x)
Supplement: Supplementary file 1 — Supplementary file1 (DOCX 109 KB) [file 40618_2024_2354_MOESM1_ESM.docx]

**Supplementary material**

**Journal of Endocrinological Investigation**

**Title**

*Development Of Diagnostic Algorithm For Cushing’s Syndrome: A Tertiary Centre Experience.*

**Authors**

Agathoklis Efthymiadis^1^, Helen Loo^1^, Brian Shine^2^, Tim James^2^, Brian Keevil^3^, Jeremy W Tomlinson^1^, Aparna Pal^1^ and Riccardo Pofi^1^

**Affiliations**

^1^ Oxford Centre for Diabetes, Endocrinology and Metabolism, NIHR Oxford Biomedical Research Centre, University of Oxford, Churchill Hospital, Oxford, UK.

*^2^* Department of Clinical Biochemistry, Oxford University Hospitals NHS Foundation Trust, Oxford, UK

^3^Department of Clinical Biochemistry, Manchester University Foundation Trust, Manchester Academic Health Sciences Centre, Manchester, UK

**Corresponding author**

Dr. Riccardo Pofi,

Oxford centre for Diabetes, Endocrinology and Metabolism

University of Oxford, Oxford OX3 7LJ, UK

Email: riccardo.pofi@ocdem.ox.ac.uk

**Supplementary figure 1. ROC curves of commonly used screening test for the diagnosis of Cushing’s Disease.**

**Supplementary figure 2.** ROC curves of commonly used screening test for the diagnosis of MACS.
